# Rh antigen expression during erythropoeisis: Comparison of cord and adult derived CD34^+^ cells

**DOI:** 10.4103/0973-6247.42694

**Published:** 2008-07

**Authors:** Namita Gupta, Lakshmi Kiran Chelluri, Kamaraju Suguna Ratnakar, K. Ravindhranath, A. Vasantha

**Affiliations:** *Transplant Immunology and Stem cell Laboratory, Global Hospitals, Hyderabad, India*

**Keywords:** Band 3, CD34 positive cells, CD47, erythropoeisis, glycophorin A, hematopoeitic stem cells, Rh polypeptides, RhAG

## Abstract

**Objectives:**

Concentrations of O_2_ and CO_2_ in the fetal circulation differ to that in maternal blood. Previous studies done in algae demonstrate the functional role of Rh antigen as CO_2_ transporter. As a preliminary study, it was the aim of this project to compare the expression of Rh polypeptides on cord and adult red blood cell progenitors during ex vivo proliferation and differentiation of CD34^+^ cells during erythropoeisis.

**Materials and Methods:**

CD34 positive hematopoeitic progenitor cells were isolated from umbilical cord blood and adult peripheral blood using an immunomagnetic system and cultured in serum free medium containing erythropoietin in order to compel them along the erythroid lineage. Cultured cells were analyzed for cell surface marker expression by flow cytometry, using monoclonal antibodies to RhAG, Glycophorin A, Rh polypeptides, CD47 and Band 3. Cytospin analysis was also done to study the morphology of cultured cells.

**Results:**

The appearance of cell surface markers analyzed on different days of culture varied slightly between samples. There was no evidence to suggest that RhAG, GPA, CD47 and Band 3 expression was any different between adult and cord derived cells. Nevertheless, the results of Rh antigenic expression suggest a reasonable difference between the two groups with adult sample derived cells showing higher and earlier expression than cord blood derived cells. These preliminary findings require further investigation.

**Conclusion:**

Comparing the expression of cell surface markers especially Rh polypeptides between adult and cord blood derived erythroid progenitors might assist in discerning their functions and could be valuable in the study of erythropoeisis.

## Introduction

**Figure 1:**
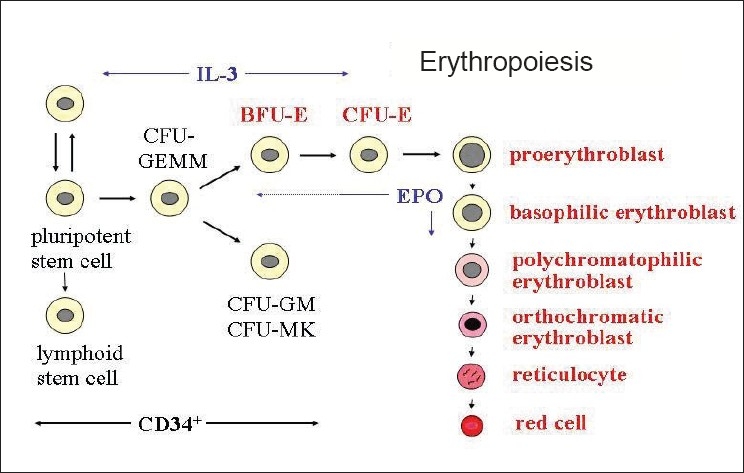
Development of mature red cell from pluripotent stem cell in presence of cytokines (BFU-E: Burst-forming unit erythroid, CFU-GEMM: Colony forming unit-Granulocyte Erythroid Monocyte Megakaryocyte, CFU-E: Colony-forming unit erythroid, CFU-GM: Colony Forming Unit Granulocyte Monocyte, CFU-MK: Colony Forming Unit Megakaryocyte, EPO: Erythropoeitin, IL-3: Interleukin-3)

Production of all the various blood cells from the hematopoeitic stem cells are collectively called hematopoeisis. *Ex vivo* expansion of hematopoeitic stem cells (HSCs) is the subject of intense commercial and academic interest due to their potential as a renewable source of material for cellular therapeutics.[1] Erythropoeisis encompasses the commitment of hematopoeitic stem cells to erythroid cells and the proliferation and differentiation of these erythroid cells into mature erythrocytes.[2] The process of erythropoeisis can be divided into a number of discrete parts Figure 1.[3] The early stages of erythropoeisis can be mimicked *in vitro* by isolating CD34^+^ cells and culturing them with the appropriate medium and combination of cytokines and growth factors to direct their proliferation and differentiation along the erythroid lineage.[4] Many scientists throughout the world are involved in studying antigenic expression during erythropoeisis. Bony *et al.*, found that Rh associated glycoprotein (RhAG) was the first protein detectable during the EPO-independent phase, which increased gradually whilst the RhD antigen appeared during the EPO-dependant phase.[5]

One of the long-term aims for studying erythropoeisis is to develop a technique for production of therapeutic quantities of erythrocytes from erythroid progenitors. The potential benefit of this development would be the supply of unlimited amount of red blood cells for transfusion, which would be safer than the use of donor blood for erythrocyte transfusion.[6]

Antigens of the Rh blood group system are products of *RHD* and *RHCE* genes.[7,8] Regulation of the expression of the *RH1* gene of *Chlamydomonas reinhardtii* by CO_2_ availability provides experimental evidence that Rh proteins, including those of humans, may be biological gas channels for CO_2._ It was found that expression of *RH1* was high for cells grown in air supplemented with 3% CO_2_ or shifted from air to high CO_2_ (3%) for three hours which suggests that Rh proteins can act as gas channels for CO_2_.[9,10]

The possibility that CO2 does not simply diffuse through the bulk lipids within membranes came from recent experiments where an aquaporin blocker inhibited CO_2_ uptake by *Synechococcus* sp. and addition of 4, 4’-diisothiocyanato-stilbene-2, 2’-disulphonate to RBCs inhibited the Cl^-^/HCO_3_^-^ exchanger (band 3) in the RBC membrane.[11,12] It has also been shown that band 3 and Rh are closely associated, forming a single macrocomplex that may function as an integrated CO_2_/O_2_ gas exchange unit in the red blood cell membrane.[13]

The aim of this study was to compare the expression of Rh antigen on cord and adult red blood cell progenitors during *ex vivo* proliferation and differentiation of CD34^+^ cells.

Structures of the Rh polypeptides have a similarity to membrane transporters. A recent study done by Ripoche *et al.*, shows that RhAG facilitates CH_3_NH_2_/NH_3_ movement across the RBC membrane and represents a potential example of a gas channel in mammalian cells.[14,15] Rh is a ubiquitous protein and studies of the biological role of Rh in a green alga suggest a role in transporting CO_2_ across the cell membrane. It has also been proposed that a band 3 based complex including Rh, RhAG, GPA, GPB, CD47 and LW may function as an O_2_/CO_2_ gas exchange unit.

Concentrations of O_2_ and CO_2_ in the fetal circulation differ to that in maternal blood. As a preliminary study, it was the aim of this project to compare the expression of Rh polypeptides, GPA, RhAG, band 3 and CD47 on CD34^+^ cells isolated from cord and adult blood during erythropoeisis. CD34^+^ cells were cultured in a serum free medium to which EPO, IL-3 and SCF had been added and tested with antibodies to cell surface markers as they differentiate.

## Materials and Methods

### Collection of samples

Cord blood samples were obtained from Dr. Denning-Kendall (Dept. of Transplantation Sciences, University of Bristol) after taking consent from the respective mothers. Ethical approval for the samples was taken from the local ethical committee. Prior IRB (Institutional Review Board) approval has been taken for the study. Blood from clamped umbilical cords were collected into a sterile 50 ml tube containing 1000 i.u. heparin. Buffy coats were obtained from the Processing, Testing and Issue department of the National Blood Service, Bristol.

### Isolation of CD34^+^ cells from cord blood and adult peripheral blood

Blood samples were diluted with Hanks Balanced Salt Solution (HBSS) and overlayed onto an equal volume of Histopaque. After centrifugation, interface cells were removed and washed in an excess of HBSS. The cells were then treated with cold sterile red cell lysis buffer to hemolyse any erythrocytes present. A mononuclear cell count was done in a Neubauer counting chamber. CD34 positive cells were isolated from the mononuclear cells using immunomagnetic positive selection method. The target cells were magnetically labeled with MACS Microbeads and were incubated with the anti-CD34 antibody, then run through a column where the CD34 cells were retained by a strong magnet. Then columns were removed from the magnet and the retained cells were flushed through as the enriched, positively selected cell fraction. Finally a CD34 positive cell count was performed and the cells re-suspended in complete media to a concentration of 1×10^5^ cells/ml.

### Culture of CD34^+^ cells in serum free medium containing EPO

CD34 positive cells were cultured in STEMSPAN™ Serum free Expansion medium (StemCell Technologies) supplemented with 10 ng/ml SCF (stem cell factor) [R and D Systems], 3 IU/ml EPO (erythropoietin) [Roche], 40µg/ml LDL (low density lipoprotein) [Calibochem], 1 ng/ml IL-3 [RandD Systems], 0.1ng/ml Prograf FK-506 [Fujisawa, Killorglin, Ireland] and PenStrep (Penicillin and streptomycin) [Sigma]. The cultures were incubated at 37°C in 5% CO_2_ in humidified air and fresh medium (with fresh cytokines) was added almost daily.

The cell concentrations were maintained at 1×10^5^ cells/ml and were monitored daily by doing cell counts using a Neubauer counting chamber.

### Analysis of cultured cells by flow cytometry

Cells cultured from both the cord blood and buffy coat samples were analyzed for their cell surface antigen expression by flow cytometry using a Becton-Dickenson FACSCalibur on days 4, 5, 6, 7, 8, 11 and 12 where possible. The primary mouse anti-human antibodies used were LA1818, BRIC256, BRIC69 and BRIC126 and BRIC 6 specific for RhAG, GPA, Rh, CD47 and Band 3 respectively.

Approximately 3 × 10^5^ cells were required per test for flow cytometry. The cells were pelleted, washed and re-suspended in PBSA (phosphate buffered saline with 1% bovine serum albumin) with 6% rabbit serum [Dako] (acts as FcR blocker). The cells were then incubated with supernatant monoclonal mouse anti-human primary antibodies for 30 minutes at room temperature. The cells were next incubated with F (ab’)_2_ fragment of rabbit anti-mouse globulins phycoerythrin (PE) conjugated (secondary) in the dark for 30 min at 4°C. The cells were finally washed and suspended in PBSA and the results were analyzed using CellQuest software [Becton-Dickenson]. The percentage gated value indicates the proportion of gated cells positive (i.e. in the M1 region, greater than 10^1^) for a particular antigen, while the geometric mean is quoted to give an indication of the level of fluorescence.

### Preparation of Cytospin slides for microscopic analysis

Cytospin slides were prepared to study the morphology of the developing cells. For cytospins, 2 × 10^4^ cells per sample were required in a total volume 300µl 1% filtered PBS-A. The samples were spun onto the cytospin slides for 5 minutes at 1350 rpm using a Shandon Cytospin 3 centrifuge. The slides were then air dried, fixed in methanol followed by staining with neat May-Grunwald stain, washing in Sorensons Buffer and incubation in Giemsa stain [1:20]. Slides were rinsed with water and allowed to dry before analysis to study the morphology. Images of the cells were recorded using an Olympus Camedia digital camera.

## Results

### Collection of samples

Different volumes of blood collected from the buffy coat and cord blood samples are shown in Table 1. Mononuclear cell counts and CD34 positive cell counts done on each sample are as shown in Table 2. All but two of the samples showed sufficient proliferation over the days monitored to perform flow cytometric analysis (A260505N and A020605A).

**Table 1:** Information of blood samples used for the project, supplied by the National Blood Service, Bristol

Date	Sample name	Unit number/Sex	Volume	Rh phenotype
12/5/2005	C120505	Female (born on 12/5/05)	35 ml	R1r
12/5/2005	A120505	GO52 505 207 188 Z	52 ml	rr
19/5/2005	C190505	Female born on 16/5/05	35 ml	rr
19/5/2005	A190505	G052 505 184 182 E	56 ml	R1R2
26/5/2005	A260505	G052 505 215 768 W	59 ml	R1R1
2/6/2005	A020605B	G052 505 224 680 W	48 ml	R1r
2/6/2005	A020605A	G052 505 113 377 A	52 ml	R2R2 or R2r
2/6/2005	C020605	Male born on 31/5/05	30 ml	R1r
9/6/2005	A090605	G052 505 234 267 Q	54 ml	R1r
9/6/2005	C1090605	Female born on 9/6/05	20 ml	R1R2
9/6/2005	C2090605	sex and date unknown	30 ml	R1r

First letters ‘A’ and ‘C’ in sample names represent Buffy coats and cord samples respectively

**Table 2:** The mononuclear and CD34^+^ cell counts obtained from cord and adult samples

Date	Source	Mononuclear cell count	CD34^+^ cell count
12/5/2005	Cord blood	8.1 × 10^7^	5.4 × 10^5^
12/5/2005	Buffy coat	7.95 × 10^8^	6.8 × 10^5^
19/5/2005	Cord blood	2.48 × 10^8^	5.4 × 10^5^
19/5/2005	Buffy coat	5.6 × 10^8^	4 × 10^5^
26/5/2005	Buffy coat	8.52 × 10^8^	4.6 × 10^5^
2/6/2005	Buffy coat(B)	13.86 × 10^8^	4.6 × 10^5^
2/6/2005	Buffy coat(A)	6.5 × 10^8^	2.5 × 10^5^
2/6/2005	Cord blood	1 × 10^8^	4.68 × 10^5^
9/6/2005	Buffy coat	8.1 × 10^8^	7.6 × 10^5^
9/6/2005	Cord blood (1)	0.8 × 10^8^	6.08 × 10^5^
9/6/2005	Cord blood(2)	6.8 × 10^8^	3.68 × 10^5^

### Analysis of cell surface marker expression by flow cytometry

Cell counts were carried out on days 4, 5, 6, 7, 8, 11 and 12 of the samples cultured and flow cytometric analysis for cell surface antigen expression was performed. The cell surface antigens studied were RhAG, GPA, Rh, CD47 and Band 3. Percentage gated (% of cells positive for the antigen) and Geometric mean (level of fluorescence, depending on the antibody binding and copy numbers of the antigen) were the two measures taken to summarize the results.

### Within sample comparisons for the expression of cell surface markers

**Figure 2:**
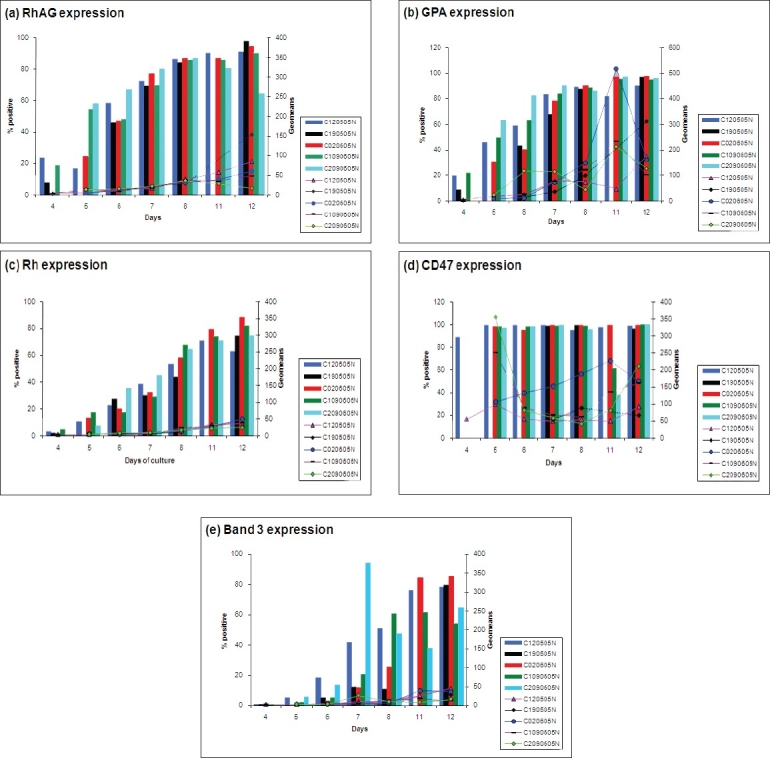
Comparisons within the cord samples cultured on 12/05/05, 19/05/05, 02/06/05 and 09/06/05. The cell surface antigens investigated were (a) Rh associated glycoprotein (b) Glycophorin A (c) Rh protein (d) CD47 (e) Band 3. Dotted lines represent extrapolated data when there were not enough cells to carry out flow cytometry. The bar chart represents the percentage of positive cells in M1 region of histograms while the line graphs represent the geometric means

Proteins involved in the Rh complex of the erythrocyte membrane were selected for this study. Cell number and availability of antibody were limiting factors. However, the results obtained show that there is no discernable difference in protein expression due to different Rh phenotypes. Comparison within cord samples for the antigens studied is shown in Figure 2a-e.

It is apparent that minor variations were present in cell surface markers’ expression and geomeans on the cells derived from cord samples cultured on different days. Rh protein shows the least amount of variation in geomeans within samples. There is a much higher level of variation in geomeans with CD47 and GPA antigens. Percentage positive cells in CD47 seem to be very high in all samples throughout the culture whilst RhAG and GPA started off low but increased by about day 5. A gradual increase can be seen for Rh expression over the culture period in all the cord samples. There is a huge variation in Band 3 expression within cord samples throughout the culture period. Geomeans for RhAG are very similar until day 8 in all the cord samples studied, starting from low and increasing gradually over the period of culture. Day 8 onwards, slight differences were observed within the samples. In contrast, huge variations can be seen in geomeans for GPA and CD47 within the samples throughout the culture period. Geomeans for Rh and band 3 are very similar in all the cord samples, starting off from low and reaching to about 30% on an average on day 12.

**Figure 3:**
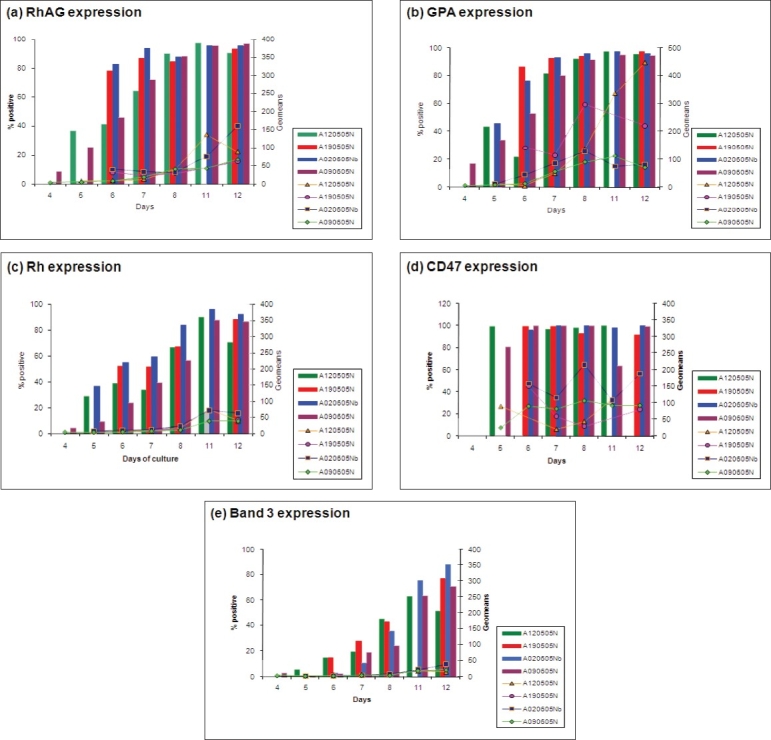
Comparisons within the adult samples cultured on 12/05/05, 19/05/05, 02/06/05 and 09/06/05. The cell surface antigens investigated were (a) Rh associated glycoprotein (b) Glycophorin A (c) Rh protein (d) CD47 (e) Band 3. Dotted lines represent extrapolated data when there were not enough cells to carry out flflow cytometry. The bar chart represents the percentage of positive cells in M1 region of histograms while the line graphs represent the geometric means

Comparison within adult samples for the antigens studied is shown in Figure 3a-e.

Comparison of the adult samples shows a similar trend to the cord samples. Again adult samples are very similar in percentage positive in all samples, at least showing the same trend if nothing else. Again band 3 shows most variation. Geo means are also very similar in the adult samples with the exception of CD47 and GPA where there is a higher variation.

### Adult and cord samples comparison for the expression of cell surface markers

**Figure 4:**
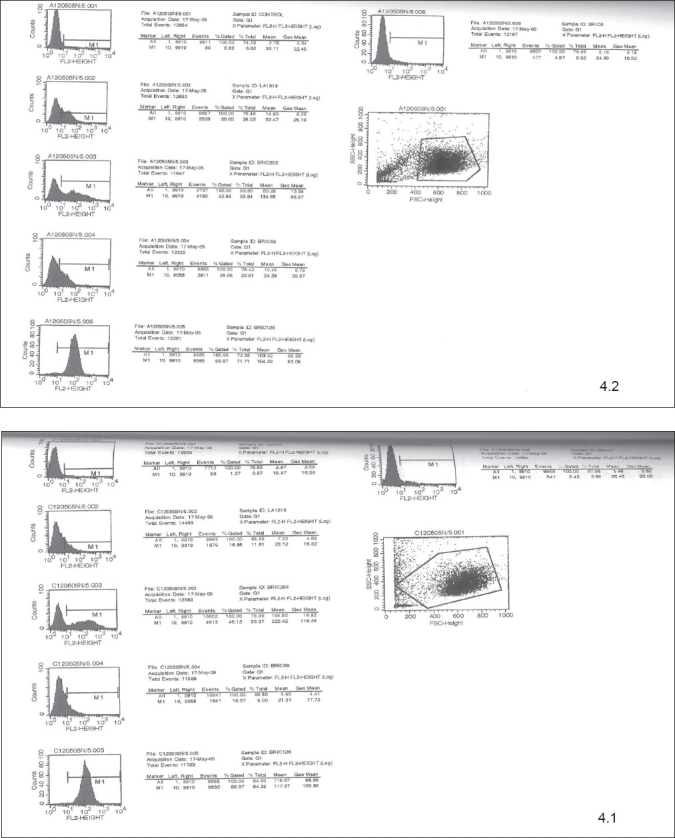
Flow Cytometry pictures for the analysis of cell surface marker expression for the adult (4.2) and cord (4.1) blood samples cultured on 12/05/05 on day 5

**Figure 5:**
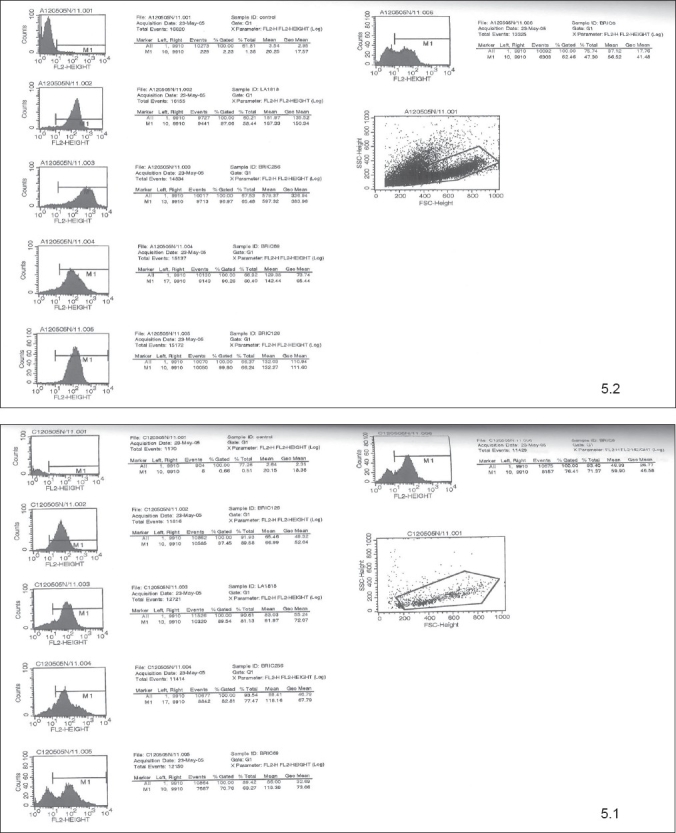
Flow Cytometry pictures for the analysis of cell surface marker expression for the adult (5.2) and cord (5.1) blood samples cultured on 12/05/05 on day 11

To compare the adult and cord blood derived cells for the expression and mean fluorescence intensity of the cell surface markers, 9 samples were studied (4 adult and 5 cord samples). The Flow Cytometry pictures for the analysis of cell surface marker expression are shown for the adult and cord blood samples cultured on 12/05/05 on day 5 and 11 as an example in the Figures 4 and 5.

**Figure 6:**
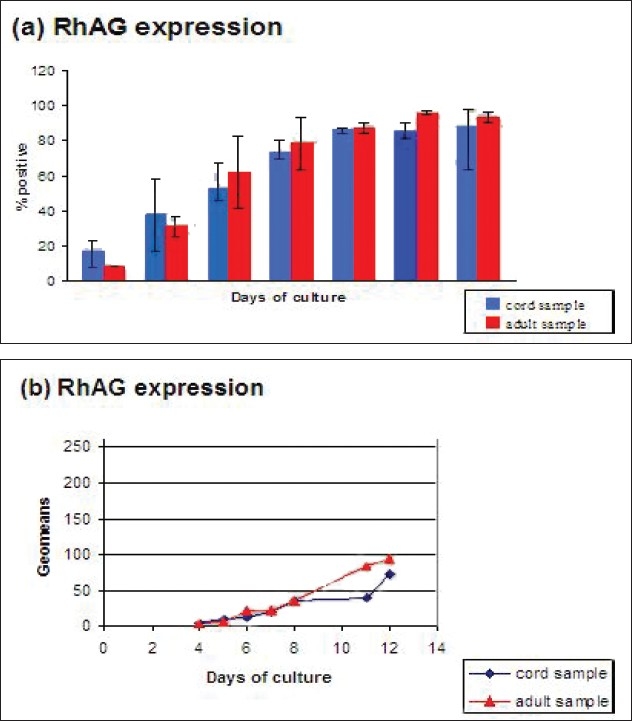
Comparison between adult and cord samples for percentage of cells positive for RhAG (a) and mean fluorescence intensity (geomeans) for RhAG (b). Error bars in 6(a) represents the variation of average values from the individual values of the samples studied

**Figure 7:**
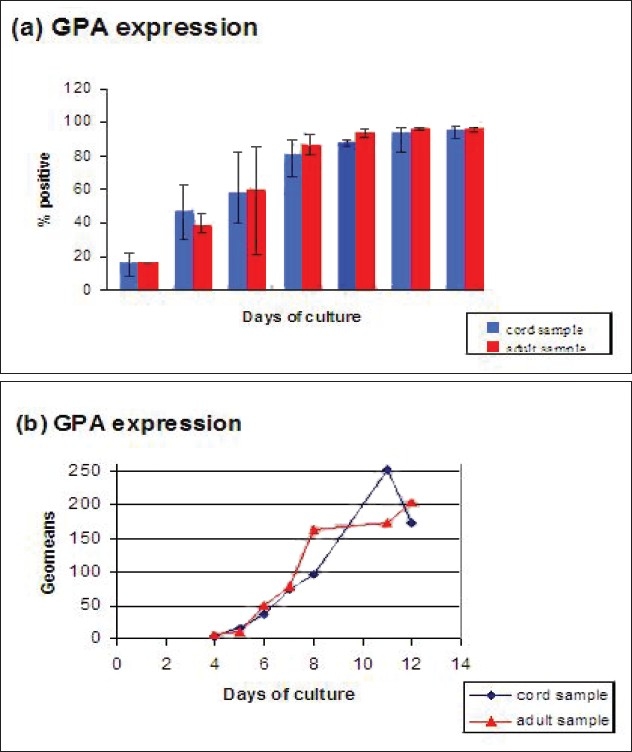
Comparison between adult and cord samples for percentage of cells positive for GPA (a) and mean flfluorescence intensity (geomeans) for GPA (b). Error bars in 7(a) represent the variation of average values from the individual values of the samples studied

**Figure 8:**
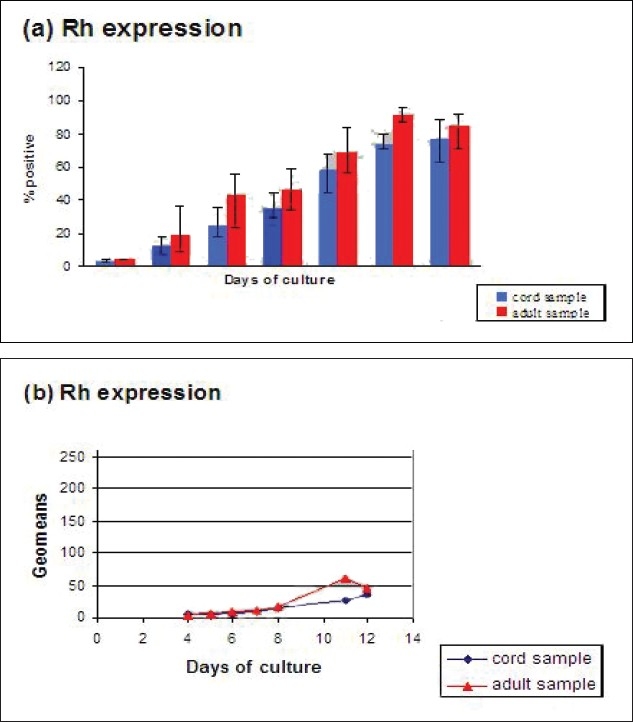
Comparison between adult and cord samples for percentage of cells positive for Rh (a) and mean flfluorescence intensity (geomeans) for Rh (b). Error bars in 8(a) represent the variation of average values from the individual values of the samples studied

**Figure 9:**
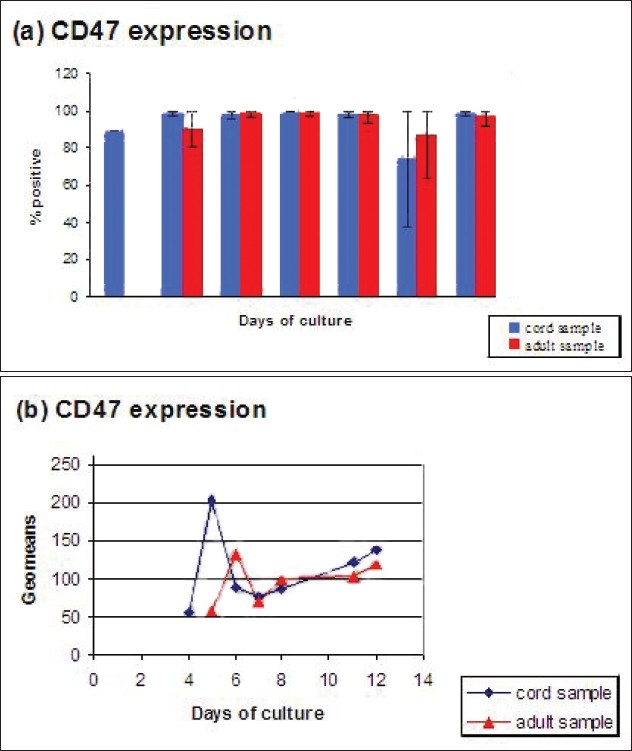
Comparison between adult and cord samples for percentage of cells positive for CD47 (a) and mean flfluorescence intensity (geomeans) for CD47 (b). Error bars in 9(a) represent the variation of average values from the individual values of the samples studied

**Figure 10:**
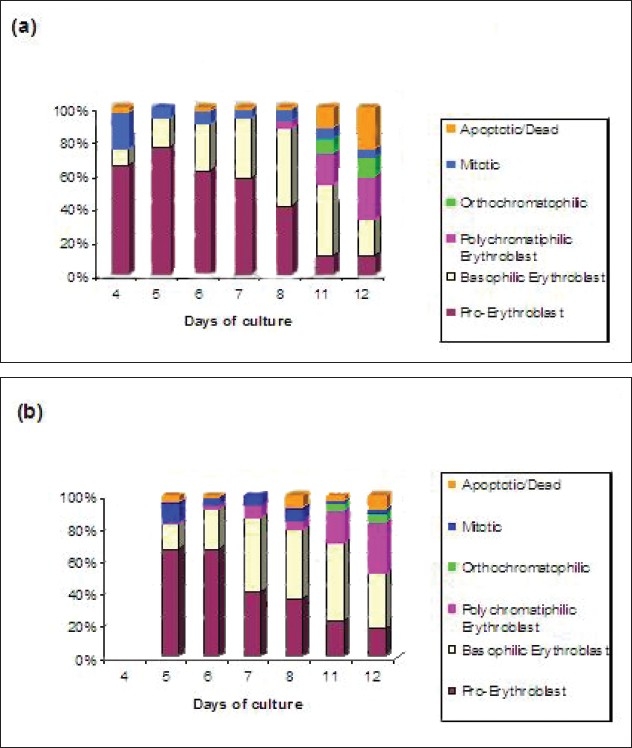
Comparison between adult and cord samples for percentage of cells positive for Band 3 (a) and mean flfluorescence intensity (geomeans) for Band 3 (b). Error bars in 10(a) represent the variation of average values from the individual values of the samples studied

Figures 6–10 show cell surface markers expression differences between adult and cord samples.

As shown in Figure 6a, both the adult and cord samples show a gradual increase in %+ cells for RhAG antigen over the duration of the culture. The cells derived from cord samples appear to have higher RhAG expression on the initial days of culture. While adult samples seem to have higher percentage of cells positive for RhAG from day 6 onwards. The geometric means for both are very similar until day 8, after which adult samples show higher values on days 11 and 12 Figure 6b. The general trend of both the adult and cord samples is that there is an increase in the geomeans. Therefore, as the cells differentiate, more fluorescently conjugated antibody binds to the cells, indicating that there is more of the antigen expressed per cell as time progresses.

From Figure 7a, GPA expression of the cells derived from cord and adult samples increased steadily over the culture period. It shows a similar trend to that of RhAG, with cord samples having higher percentage of positive cells until day 5. But the error bars are very large at these points, so whether this trend exists, is not very certain. Adult samples have higher expression levels when compared to cord samples on rest of the days of culture, although this difference is very marginal. In contrast, the geometric means show a general trend of increasing until day 11 for both the samples Figure 7b. Cord samples have generally lower values than adult samples throughout the culture period except on days 5 and 11 when cord samples have slightly greater geomeans.

Figure 8a shows a gradual increase in Rh expression for both cord and adult samples. Rh expression shows a very consistent trend, with adult samples having higher percentage of positive cells over the culture period when compared to the cord samples. On average, adult samples also show earlier expression of Rh protein than cord samples.

The geometric means of adult and cord samples follow a similar trend to the percentage of cells positive for Rh Figure 6b. Overall, the % positive and geomean values for Rh protein are relatively at a lower level as compared to RhAG and GPA, indicating that the cells express relatively lower Rh protein and there are lower copy numbers of Rh antigen.

The trend for CD47 expression is very similar for the cells derived from both adult and cord samples throughout the period of flow cytometry study Figure 9a. Also the trend for geometric means seems pretty similar for both adult and cord samples in that they both start low, show a sharp increase (higher in the cord than adult) followed by a sharp drop which then increases steadily to similar levels by the end of the cultures except that the cord samples seem to be slightly ahead of the adult samples Figure 9b.

Band 3 expression increases over the course of the culture in both cord and adult samples Figure 10a. Cord samples generally show slightly higher band 3 expression when compared to adult samples, with the exception of days 4 and 11. There is no major difference observed between the geo means of the adult and cord samples Figure 10b. There is a gradual increase in the geo mean of band 3 for both adult and cord samples over the culture period.

An arbitrary cut off point of 30% ± 1% was taken to determine if there was any difference in cell surface markers' expression between adult and cord samples. This cut off was selected as most of the samples reach 30% ± 1% positive cells fairly early in the culture, therefore comparisons can be made easily. CD47 expression could not be included as all the samples show a high level of expression for the CD47 antigen from very early in the culture which is maintained throughout the culture period. The antigens studied were RhAG, GPA, Rh protein and band 3. Data for sample A190505 could not be obtained for day 5 but % positive cells for RhAG, GPA and Rh are high on day 6, so it was assumed that >30%±1% cells are positive for these antigens by day 5. The data presented in Table 3 show that all the samples, both cord and adult are >30%±1% cells positive for RhAG expression on either day 5 or day 6 and for GPA expression on day 5, with the exception of cord sample C190505 which shows >30%±1% cells positive for GPA on day 6. All the adult samples, with the exception of A090605, show >30%±1% cells positive for Rh expression as early as day 5 whereas cord samples do not reach >30%±1% cells positive until day 7 apart from C02090605. So, it seems that there is a time elapse of 2 days between adult and cord samples for Rh antigen expression. No conclusive data could be obtained for band 3.

**Table 3:** Time elapsed (days) for the samples to have >30%±1% cells positive for the antigenic expression

Samples	RhAG	GPA	Rh protein	Band 3
A120505N	Day 5	Day 5	Day 5	Day 8
A190505N	Day 5	Day 5	Day 5	Day 8
A020605Nb	Day 6	Day 5	Day 5	Day 8
A090605N	Day 6	Day 5	Day 7	Day 11
C120505N	Day 6	Day 5	Day 7	Day 7
C190505N	Day 6	Day 6	Day 7	Day 11/12
C020605N	Day 6	Day 5	Day 7	Day 11
C1090605N	Day 5	Day 5	Day 7	Day 8
C2090605N	Day 5	Day 5	Day 6	Day 7

### Cytospins

Cytospin slides were prepared on almost all days on which flow cytometry was performed. Any morphological variation between adult and cord sample cultures could then be directly compared to the differences observed from the flow cytometry results.

The percentages of various cell types were calculated by counting the cells by light microscopy. Data in Figurs 11 and 12 are constructed from raw data obtained (not shown) by microscopic analysis of the cytospin preparations of cells derived from adult and cord samples cultured on 2/06/05 and 9/06/05 respectively. Data from other samples is not shown as the samples followed a similar trend as far as distribution of cell types is concerned on the days of culture studied.

**Figure 11:**
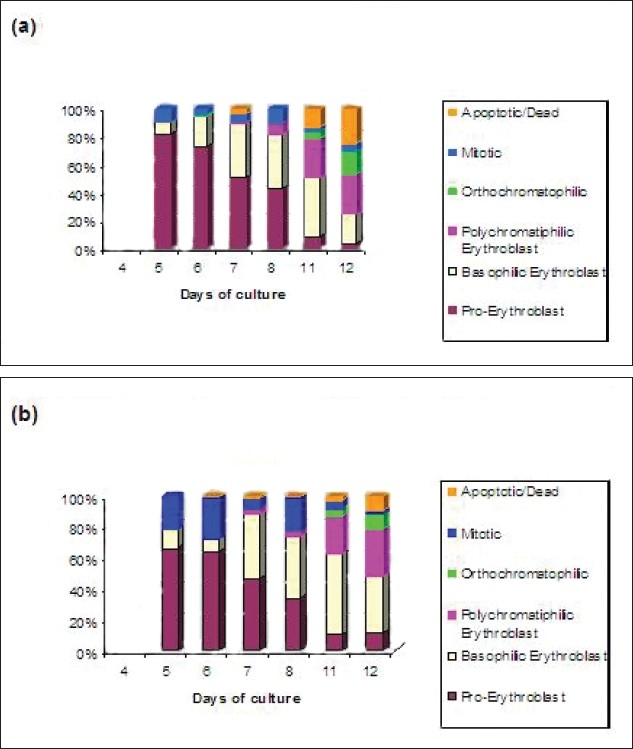
Cytospin results of adult sample (A020605) (a) and cord sample (C020605) (b). Identifified cell types studied were Pro-Erythroblast, Basophilic Erythroblast, Polychromatophilic erythroblast and Orthochromatophilic Erythroblast, Mitotic and Apoptotic cells

**Figure 12:**
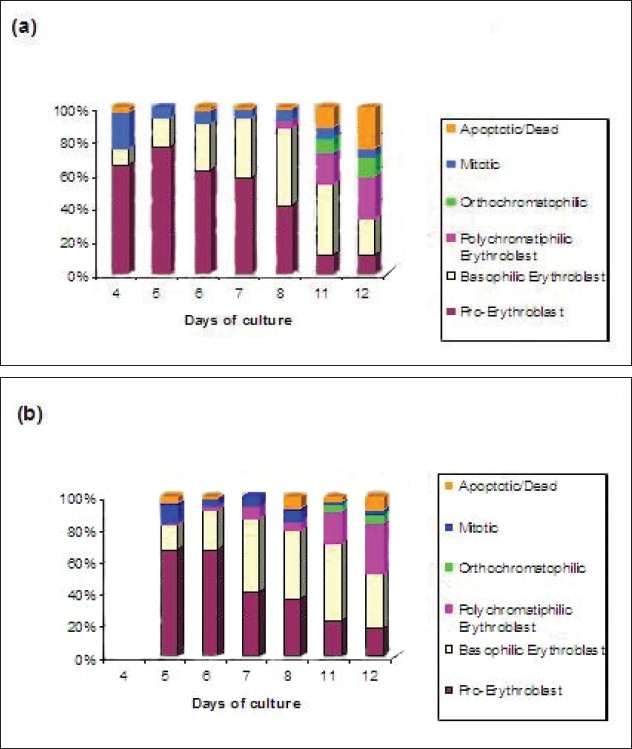
Cytospin results of adult sample cultured on 09/06/05 (a) and cord sample 2 cultured on 09/06/05 (b). Cell types studied were Pro-erythroblast, basophilic erythroblast, polychromatophilic and orthochromatophilic erythroblast, mitotic and apoptotic cells

Some obvious differences can be seen between the cells cultured from adult and cord samples Figure 11. On day 5, the adult sample has 10% cells mitotic as compared to 21% in the cord sample, indicating that cells from cord sample are proliferating faster than cells from adult sample. By day 6, the adult sample has almost 2.5 times more basophilic erythroblast cells than cord sample (21% and 8% respectively). This indicates that cells from adult sample are differentiating at a faster rate when compared with cord sample. Similarly, on day 8, there are more polychromatophilic erythroblasts in the adult sample than the cord sample (7% and 3% respectively). By day 12, there is higher percentage of orthochromatophilic and apoptotic/dead cells in adult sample than cord sample (16% and 25%; 9% and 9% respectively).

Figure 12 shows the cytospin results of samples cultured on 9/06/05.

The cord sample C2090605 had comparatively more number of polychromatophilic erythroblasts on days 6 and 7 when compared to the adult samples. Overall, in spite of slight variations between different samples, it was consistently found that the cells cultured from adult samples die early as compared to cord samples as was obvious from the flow cytometry results. The only real difference that could be seen between the samples was that the cord sample C020605 had more mitotic cells early and less dead cells and with the adult samples there are no major differences.

**Figure 13:**
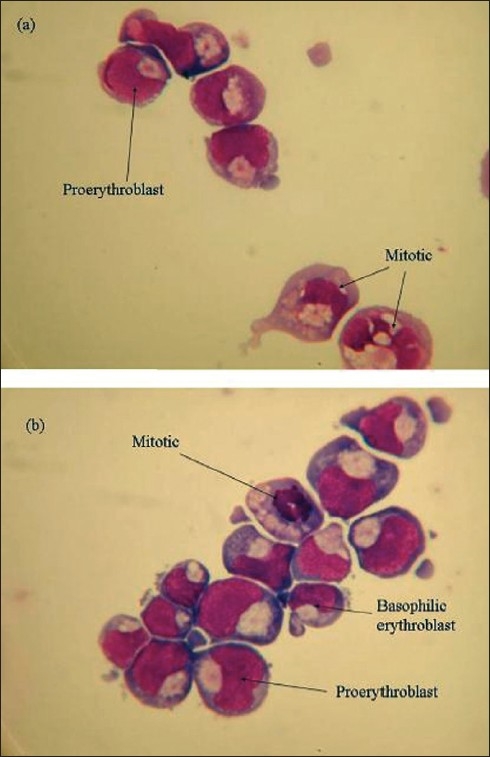
Images of cytospin slides on day 6, of cells derived from cord sample, C020605 (a) and adult sample, A020605 (b)

**Figure 14:**
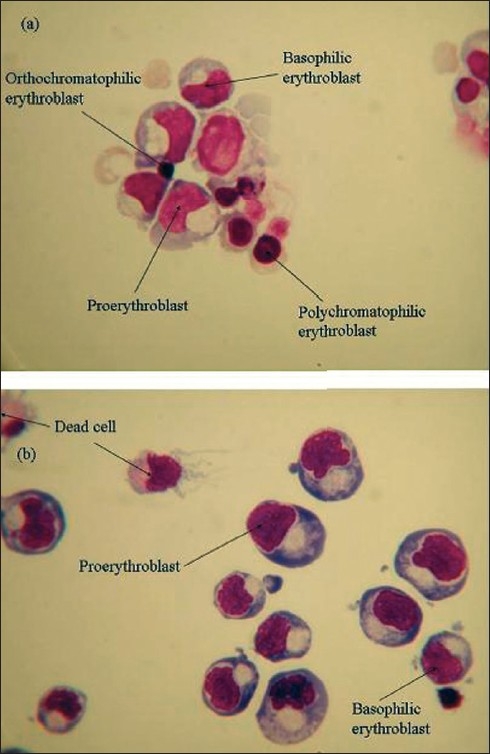
Images of cytospin slides on day 8, of cells derived from adult sample, A020605 (a) and cord sample, C020605 (b)

**Figure 15:**
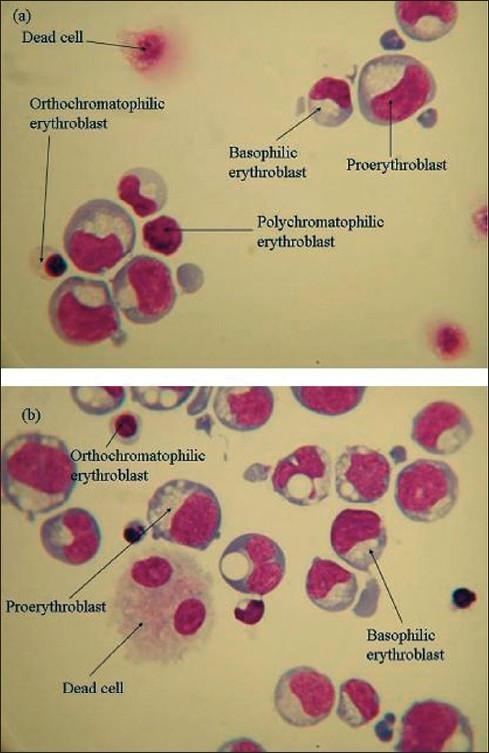
Images of cytospin slides on day 12, of cells derived from adult sample, A090605 (a) and cord sample 1, C1090605 (b)

The images of cytospin slides on days 6, 8, 11 and 12 from cord and adult samples cultured on 2/06/05 and 9/06/05 are shown as representative examples of cell types observed Figures 13–15.

## Discussion

The aims of this project were to determine if there were differences in Rh antigen expression between the adult and the cord blood derived CD34 positive cells cultured along the erythroid lineage *in vitro*. The expression of RhAG, GPA, Rh, CD47 and band 3 antigens were compared using flow cytometry and cell morphology was studied using cytospin staining. A total of 9 samples were studied (5 cord and 4 adult samples).

Minor variations in the level of antigenic expression were observed in cultures derived from different individuals. This may be attributed to various biological factors such as age, sex, stress and health. Static cell cultures have been used for the present study which involves maintenance of cell concentrations and addition of cytokines manually but as the cytokine microenvironment in such cultures is dynamic and proliferation is constantly occurring, it is highly challenging to optimize the cytokine composition in *ex vivo* culture medium. The problem of such static culture could be overcome by a continuous flow culture, where the medium is constantly being cycled and so the nutrients are replenished and waste products are removed without any build-up of growth factors that can influence the culture output.[1]

All the samples showed a very similar trend for RhAG, GPA, Rh and CD47 expression. While marked differences were observed within the cord samples for band 3 expression especially with the sample C2090605. Similarly geomeans for all the antigens followed a similar trend within the cord and the adult samples except for GPA and CD47. For e.g. the cord sample C2090605 showed comparatively higher geomeans for GPA on days 6 and 7 as compared to other samples. On correlating these results with the microscopic analysis, it was found that the cells derived from this sample were further along the erythroid lineage than other samples on the same days. As more mature cells have a higher copy number of GPA molecules per cell this would explain the observed difference in geomeans in this sample. Similarly the cord sample C020605 showed extraordinarily higher geomean for GPA on day 11. These variations can be explained because GPA has a very wide variation in copy number per cell (i.e. between 3 and 12 × 10^5^ ) compared to the smaller range for Rh (1-2 × 10^5^ molecules per cell).[16]

The intra-sample variations observed might be due the small number of samples studied which make the differences more apparent than if a larger number of samples were studied.

Analysis of the cell morphology using cytospins stained with May-Grunwald and Giemsa consistently showed that all the samples show very similar trend with regard to the cell types except the cord sample C2090605 mentioned above. The cells cultured from adult samples underwent cell death earlier than the cord samples. This was in accordance with the flow cytometry data with antigen expression dropping slightly towards the end of the cultures.

As far as the adult and the cord samples comparison is concerned, RhAG antigenic expression followed a similar trend in both, starting with low expression and then gradually increasing over the culture period. The adult samples had slightly higher RhAG expression than the cord samples throughout the culture except on initial days.

Mean fluorescence intensity (geomean) for RhAG was very similar in both the adult and the cord samples until day 8 after which the values for adult samples were higher on subsequent days.

GPA expression also followed the same trend as RhAG in both the adult and the cord samples. Geomeans for both the samples were similar until day 7 after which the adult samples have higher values except on day 11. As shown in the results, no relevant difference could be observed between the adult and the cord samples in time elapsed (days) for the samples to reach >30%±1% cells positive for the RhAG and GPA antigenic expression. Therefore it is very difficult to make any conclusion regarding differences in RhAG and GPA expression between the adult and the cord samples derived cells.

CD47 expression and geomeans were also very similar in both the adult and the cord samples. Band 3 expression followed the same pattern in both the adult and the cord blood derived cells, starting with lower expression and increasing gradually over the culture although the cord samples had slightly higher expression than the adult samples throughout the culture. Geomeans were very similar for both the samples.

As far as Rh expression is concerned, there may be some difference between the adult and the cord blood derived cells. From the results obtained, it seemed that there is not a marked difference due to samples with different Rh phenotypes. The adult samples derived cells had higher expression on all the days of culture when compared to the cord blood derived cells. There was a reasonable difference of 2 days between the adult and the cord samples with adult samples having earlier expression, when time elapsed for the samples to reach >30%±1% cells positive for Rh was considered. Geomeans for both the samples also followed the similar trend except on day 11, when the adult samples had higher values. Cell death increased on day 12 for most of the adult samples studied which is also obvious from the cytospin results. This is because the cells no longer have all the growth factors or cytokines and macrophages required for terminal differentiation into fully mature erythrocytes. As there was not a huge difference observed amongst the samples for Rh expression, it may be possible that the difference in Rh expression between the adult and the cord samples is actually a true difference. This difference cannot be deemed significant due to the small sample number in this study. However with more time and a larger sample group this is an interesting result that would warrant further investigation. Statistical tests are required which would give more weight to the observations.

Another important finding of the present study is that the MFI for Rh and Band 3 antigens on all the samples studied were very similar over the culture period. This finding supports the view that band 3 and Rh complex are associated in the RBC membrane which has been studied earlier.[13,17]

In accordance with the previous report by Suyama *et al.* and Nicolas *et al.*, here also it was found that Rh and RhAG are expressed in parallel. Both RhAG and Rh showed 30% cells positive by days 5 or 6 although the overall expression for Rh throughout the culture period remains at a relatively lower level than that of RhAG.[7,18]

Although some tentative conclusions about the differences in adult and cord blood can be drawn from the results presented in this study, there are some limitations to the techniques used which could have influenced an overall pattern of results obtained.

The differences between the adult and cord blood samples observed might have been due to different anticoagulants used when the blood was collected. The adult samples were collected into blood packs containing chemical anticoagulant, CPD (citrate phosphate dextrose) while the cord samples were collected using a biological anticoagulant, heparin. The standardization of these collection methods would allow the samples to be more directly compared and the observed difference then could have more weight. Moreover, information on the time of collection of the samples was not always provided, which could have some influence on the initial cell number and the general health of the cells. To compare adult and cord samples for statistical analysis, potential variables should be eliminated from the study as much as possible to avoid the bias. All cultures should undergo the same treatment at all stages from collection and isolation to culture and testing.

A factor that was not addressed in this study is that the adult and cord samples were cultured under same carbon-dioxide concentrations i.e. 5% CO_2_. Cord blood is known to have higher *in vivo* CO_2_ concentration than adult blood; so, ideally the samples should have been cultured under different conditions with cord sample grown at a higher CO_2_ concentration than the adult samples. This was a preliminary study, so future experiments could optimize these conditions. The ultimate goal of *ex vivo* research and culture of CD34 positive cells along the erythroid lineage is their expansion for endless supplies of red cells or precursors. If we can understand how and why antigen expression changes throughout erythropoeisis and the differences between adult and cord blood, we can optimize culture conditions to maximize production of erythroid cells.

To date, the understanding of the expression of various antigens is based solely on *in vitro* studies, under very different conditions to those found *in vivo*. Therefore, further studies are required which are capable of simulating *in vivo* conditions as much as possible to allow the study of interactions amongst various antigens and their influence on expression.

In conclusion, the present work has observed an apparent difference in Rh antigenic expression between the adult and the cord blood derived erythroid progenitors, with adult derived cells having higher and earlier expression of Rh antigen than cord cells throughout the culture. Obviously, further experiments are necessary to demonstrate whether this difference is real. This may be an important finding when considering the functional role of Rh in the erythrocyte membrane.
